# Differences in attitudes towards end-of-life care among intensivists, oncologists and prosecutors in Brazil: a nationwide survey

**DOI:** 10.1186/s13054-018-2204-9

**Published:** 2018-10-26

**Authors:** João Gabriel Rosa Ramos, Roberto D’Oliveira Vieira, Fernanda Correia Tourinho, Andre Ismael, Diaulas Costa Ribeiro, Humberto Jacques de Medeiro, Daniel Neves Forte

**Affiliations:** 1 Intensive care unit, Hospital Sao Rafael, Av. São Rafael, 2152, Salvador, BA 41253-190 Brazil; 2 Palliative care team, Hospital Sao Rafael, Salvador, Brazil; 3 Clinica Florence hospice & rehabilitation center, Salvador, Brazil; 4 Federal Prosecution Service, Brasilia, Brazil; 5 Prosecution Service at Distrito Federal e Territorios, Brasilia, Brazil; 6 Federal District High Court of Appeals, Brasilia, Brazil; 7 Medical Sciences PhD Program, Faculdade de Medicina FMUSP, Universidade de São Paulo, Sao Paulo, Brazil; 8 Teaching and Research on Palliative Care Program, Hospital Sirio-Libanes, Sao Paulo, Brazil

There is great variability in end-of-life care [1] and the legal context may interfere with decisions on limitation of medical treatment [2]. In Brazil, end-of-life care was initially regulated in 2006, but legal controversies still continue [3]. Even though physicians do not need authorization from the Judiciary system to act, those controversies may cause uncertainty regarding seemingly competing professional duties (caring for patients’ best interests versus maintenance of life), possibly hampering good medical care [4]. In this study, we sought to compare the attitudes of physicians (intensivists and oncologists) and prosecutors from the Ministerio Publico da Uniao (MPU) towards common concepts in end-of-life care in Brazil, such as patient autonomy and withholding/withdrawal of care. We evaluated MPU prosecutors because they may be responsible for investigation of deaths due to limitation of medical treatment.

After ethics approval, we sent an electronic survey (SurveyMonkey Inc., USA) to intensivists, oncologists and prosecutors practicing in the 27 federative units of Brazil (see Additional file 1 for more details of methods and Brazilian judiciary and health systems). Participants were asked to rate 11 questions in a Likert scale from 1 (completely disagree) to 10 (completely agree). Responses were categorized in three groups, accordingly to the Likert scale: disagree (1–4), neutral (5–6) and agree (7–10). Categorical and continuous variables were analyzed with chi-square and Kruskal-Wallis tests, respectively, and a *p* value < 0.05 was considered as significant. Outcome was the difference in agreement between groups of respondents.

From February 2018 to May 2018 there were 661 respondents, comprising 24/27 (88.8%) federative units of Brazil, of which 467 (71%) were intensivists, 89 (13%) were oncologists and 105 (16%) were prosecutors. The characteristics of the respondents are provided in Table 1. There were significant differences in responses between physicians and prosecutors for all 11 questions, except for question 10 (Fig. 1 and Additional file 1: Table S1). Prosecutors were less likely to agree with paternalistic decision-making by physicians, more likely to agree with the maintenance of life-sustaining treatments in patients with poor prognosis and more likely to agree with the concepts of euthanasia and physician-assisted suicide, whereas physicians responded in the opposite direction.

**Table 1:** Characteristics of respondents

Characteristic	ICU	Onco	MPU	p-value
Age (years), median (IQR)	41 (35–48)	38 (34–42)	41 (35–47.5)	0.023
Years since university graduation, median (IQR)	15 (10–24)	13.5 (9–19)	18 (12–24.5)	0.003
Male gender, N (%)	246 (54.7)	39 (45.3)	78 (75.7)	< 0.001
Believes in God, N (%)	366 (81.3)	71 (82.6)	75 (72.8)	0.003
Personal experience with terminal illnesses, N (%)	402 (86.1)	68 (76.4)	77 (73.3)	0.002
Professional experience with terminal illnesses, N (%)	461 (98.9)	89 (100)	24 (22.9)	< 0.001

*ICU* intensivists, *MPU* prosecutors from the Ministerio Publico da Uniao, *Onco* oncologists

**Fig. 1:**
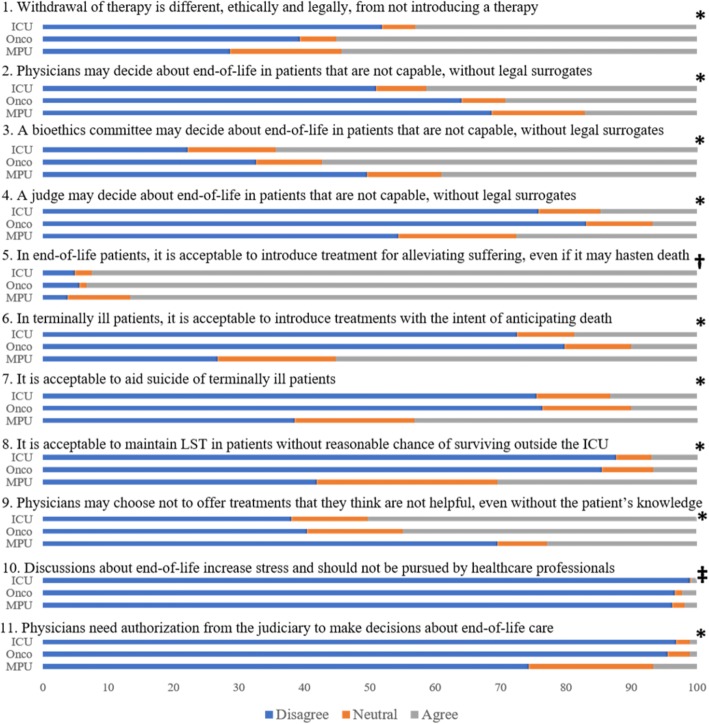
Differences in attitudes towards end-of-life care between intensivists (*ICU*), oncologists (*Onco*) and prosecutors of the Ministerio Publico da Uniao (*MPU*). Full questions are outlined in Additional file 1: Table S2. *LST* life-sustaining treatment, *ICU* intensive care unit. *p < 0.001, ^†^*p* = 0.007, ^‡^*p* = 0.183

Our results suggest that there is variation in attitudes towards end-of-life care between physicians and prosecutors. However, responses did not reflect an absolute dominance of the principle of maintenance of life over other principles. Similar variations in attitudes have been shown before [5] and may reflect professional ethics and other values. Those differences should encourage actions to reduce heterogeneity in attitudes toward end-of-life care, possibly through greater interaction between physicians and prosecutors, ensuring that patients’ wishes are respected and that clinicians are protected in their practice.

## Additional file

Additional file 1: **Table S1.** Differences in attitudes towards end-of-life care between intensivists (*ICU*), oncologists (*Onco*) and prosecutors of the Ministerio Publico da Uniao (*MPU*). Table containing supplementary data on the differences in attitudes towards end-of-life care between physicians and prosecutors. (DOCX 28 kb)

## Abbreviations

ICU: Intensivists/intensive care unit
LST: Life-sustaining treatment
MPU: Ministerio Publico da Uniao
Onco: Oncologists
