# Supplementary material for: Differences in attitudes towards end-of-life care among intensivists, oncologists and prosecutors in Brazil: a nationwide survey
Source: Crit Care. 2018 Oct 26;22:265. doi: 10.1186/s13054-018-2204-9 (PMC6204021; doi:10.1186/s13054-018-2204-9)
Supplement: Supplementary file 1 — Table S1. Differences in attitudes towards end-of-life care between intensivists (ICU), oncologists (Onco) and prosecutors of the Ministerio Publico da Uniao (MPU). Table containing supplementary data on the differences in attitudes towards end-of-life care between physicians and prosecutors. (DOCX 28 kb) [file 13054_2018_2204_MOESM1_ESM.docx]

**S1. Detailed Methods**

**S1.1. Study design**

This study was an electronic, self-administered survey. An online survey was developed electronically (SurveyMonkey Inc., USA). The survey was made available to intensivists (i.e., physicians that worked in the ICU), oncologists (i.e., physicians that worked with oncological patients, comprising clinical and surgical oncologists and hematologists-oncologists) and prosecutors from the Ministerio Publico da Uniao (MPU).

The survey was sent electronically through the AMIBnet mailing list, through the official ESMPU mailing list and through intensivists and oncologists mailing lists. The survey was available from February 2018 to May 2018.

All participants that assessed the survey and agreed to the electronic informed consent form were included in the study. It was not required that participants responded all questions of the survey and missing answers were excluded from the analysis.

**S1.2. Full questions presented to respondents**

Question 1. In your opinion, withdrawal of a therapy that has been already initiated is different, both ethically and legally, from withholding (not initiating) a therapy.

Question 2. In the event of a patient being incapable, without any advanced directives, and in the lack of agreement or absence of legal surrogates, the medical team can make decisions regarding withdrawal of treatment in end-of-life patients.

Question 3. In the event of a patient being incapable, without any advanced directives, and in the lack of agreement or absence of legal surrogates, a bioethics committee can make decisions regarding withdrawal of treatment in end-of-life patients.

Question 4. In the event of a patient being incapable, without any advanced directives, and in the lack of agreement or absence of legal surrogates, a judge can make decisions regarding withdrawal of treatment in end-of-life patients.

Question 5. In your opinion, it is acceptable to introduce medications with the intent of symptom management for patients with terminal illnesses and intense suffering, even if those medications may have side effects that could, eventually, hasten the process of dying.

Question 6. In your opinion, it is acceptable to introduce treatments with the intent of anticipating death in a patient with terminal illness and intense suffering.

Question 7. In your opinion, it is acceptable to introduce treatments with the intent or aiding suicide of a patient approaching end of life.

Question 8. In your opinion, it is acceptable that physicians may artificially sustain the life of a patient with no reasonable chance of survival outside the intensive care unit.

Question 9. In your opinion, it is acceptable that physicians may choose not to offer to a patient an alternative of treatment that the physician thinks to be harmful or non-beneficial to the patient, even if the patient has no knowledge of that alternative of treatment.

Question 10. In your opinion, conversations about end-of-life may increase stress of patients and surrogates and should not be pursued by healthcare professionals.

Question 11. In your opinion, to make medical decisions regarding the end-of-life care of a patient, physicians should need authorization from the judiciary.

**S1.3. Description of Brazilian Health System**

Brazil has a unique health system in that, although there is a Unified Health System[1], which is government-led and with no direct payment from users, there is also a private sector which covers a smaller proportion of the population[2]. Although those characteristics are unique to Brazil, other countries face similar challenges[3].

**S1.4. Description of Brazilian Judiciary System**

Briefly, in Brazil the Judiciary system is divided in two main branches, one which is local, existing in each federative unit (State Justice) and other, specialized in federal issues (Federal Justice). Each of the two branches coexists with prosecutors from the Ministerio Publico, who are responsible for criminal and civil prosecution. The survey was applied to members from the Ministerio Publico da Uniao (MPU) that act in the Federal Justice in all over the country.

Although the medical conduct is independent from the previous authorization from the Judiciary system, MPU prosecutors may initiate criminal investigations to assess deaths occurring after end-of-life decisions. Despite the fact that some aspects of end-of-life care have been regulated[4], the exact conditions for the application of those norms may not be clear, so that possible investigations against clinicians could be at the discretion of the responsible prosecutors.

**S1.5. Statistics**

Continuous data was described as median (IQR) and analyzed with Mann-Whitney U test[5]. Categorical data was described as number (%) and analyzed with chi-square test.

A two-tailed p-value less than 0.05 was considered as significant in all analyses. As suggested by others, when the data under evaluation are not random but actual observations, no adjustment for multiple comparisons was made[6]. SPSS 21.0TM (SPSS Inc., USA) was utilized as statistical software.

References

1. Paim J, Travassos C, Almeida C, Bahia L, Macinko J: **The Brazilian health system: history, advances, and challenges**. *Lancet* 2011, **377**(9779):1778-1797.

2. Bahia L, Scheffer M, Tavares LR, Braga IF: **From health plan companies to international insurance companies: changes in the accumulation regime and repercussions on the healthcare system in Brazil**. *Cad Saude Publica* 2016, **32Suppl 2**(Suppl 2):e00154015.

3. Atun R, de Andrade LO, Almeida G, Cotlear D, Dmytraczenko T, Frenz P, Garcia P, Gomez-Dantes O, Knaul FM, Muntaner C *et al*: **Health-system reform and universal health coverage in Latin America**. *Lancet* 2015, **385**(9974):1230-1247.

4. Ribeiro DC: **The right to die: the end-of-life stage in Brazil, Argentina and Colombia**. *RVMD* 2015, **9**(1):01-20.

5. Zhang Z: **Univariate description and bivariate statistical inference: the first step delving into data**. *Ann Transl Med* 2016, **4**(5):91.

6. Rothman KJ: **No adjustments are needed for multiple comparisons**. *Epidemiology* 1990, **1**(1):43-46.

Supplementary table 1. Differences in attitudes towards end-of-life care between intensivists (ICU), oncologists (Onco) and prosecutors of the Ministerio Publico da Uniao (MPU)

|  | ICU | Onco | MPU | Overall p value | ICU x Onco p value | ICU x MPU p value | Onco x MPU p value |
| --- | --- | --- | --- | --- | --- | --- | --- |
| 1. Withdrawal of therapy is different, ethically and legally, from not introducing a therapy | | | | | |  |  |
| Disagree, N(%) | 242 (51.8) | 35 (39.3) | 30 (28.6) | <0.001 | 0.092 | <0.001 | 0.029 |
| Neutral, N(%) | 24 (5.1) | 5 (5.6) | 18 (17.1) |  |  |  |  |
| Agree, N(%) | 201 (43) | 49 (55.1) | 57 (54.3) |  |  |  |  |
| Likert scale, median (IQR) | 3 (1-8) | 8 (3-8) | 7 (4-8) | <0.001 |  |  |  |
|  |  |  |  |  |  |  |  |
| 2. Physicians may decide about end-of-life in patients that are not capable, without legal surrogates | | | | | |  |  |
| Disagree, N(%) | 237 (50.9) | 57 (64) | 72 (68.6) | <0.001 | 0.068 | <0.001 | 0.056 |
| Neutral, N(%) | 36 (7.7) | 6 (6.7) | 15 (14.3) |  |  |  |  |
| Agree, N(%) | 193 (41.4) | 26 (29.2) | 18 (17.1) |  |  |  |  |
| Likert scale, median (IQR) | 4 (3-8) | 3 (3-7) | 3 (2-5) | 0.001 |  |  |  |
|  |  |  |  |  |  |  |  |
| 3. A bioethics committee may decide about end-of-life in patients that are not capable, without legal surrogates | | | | | | |  |
| Disagree, N(%) | 103 (22.1) | 29 (32.6) | 52 (49.5) | <0.001 | 0.093 | <0.001 | 0.034 |
| Neutral, N(%) | 63 (13.5) | 9 (10.1) | 12 (11.4) |  |  |  |  |
| Agree, N(%) | 301 (64.5) | 51 (57.3) | 41 (39) |  |  |  |  |
| Likert scale, median (IQR) | 8 (5-9) | 8 (3-8) | 5 (3-8) | <0.001 |  |  |  |
|  |  |  |  |  |  |  |  |
| 4. A judge may decide about end-of-life in patients that are not capable, without legal surrogates | | | | | |  |  |
| Disagree, N(%) | 354 (75.8) | 74 (83.1) | 57 (54.3) | <0.001 | 0.126 | <0.001 | <0.001 |
| Neutral, N(%) | 44 (9.4) | 9 (10.1) | 19 (18.1) |  |  |  |  |
| Agree, N(%) | 69 (14.8) | 6 (6.7) | 29 (27.6) |  |  |  |  |
| Likert scale, median (IQR) | 3 (1-4) | 3 (1-3) | 4 (3-7) | <0.001 |  |  |  |
|  |  |  |  |  |  |  |  |
| 5. In end-of-life patients, it is acceptable to introduce treatment for alleviating suffering, even if it may hasten death | | | | | | |  |
| Disagree, N(%) | 23 (4.9) | 5 (5.6) | 4 (3.8) | 0.007 | 0.689 | 0.003 | 0.037 |
| Neutral, N(%) | 12 (2.6) | 1 (1.1) | 10 (9.5) |  |  |  |  |
| Agree, N(%) | 432 (92.5) | 83 (93.3) | 91 (86.7) |  |  |  |  |
| Likert scale, median (IQR) | 10 (8-10) | 9 (8-10) | 8 (8-10) | <0.001 |  |  |  |
|  |  |  |  |  |  |  |  |
| 6. In terminally ill patients, it is acceptable to introduce treatments with the intent of anticipating death | | | | | |  |  |
| Disagree, N(%) | 338 (72.5) | 71 (79.8) | 28 (26.7) | <0.001 | 0.147 | <0.001 | <0.001 |
| Neutral, N(%) | 41 (8.8) | 9 (10.1) | 19 (18.1) |  |  |  |  |
| Agree, N(%) | 87 (18.7) | 9 (10.1) | 58 (55.2) |  |  |  |  |
| Likert scale, median (IQR) | 1 (1-5) | 1 (1-3) | 8 (8-10) | <0.001 |  |  |  |
|  |  |  |  |  |  |  |  |
| 7. It is acceptable to aid suicide of terminally ill patients | |  |  |  |  |  |  |
| Disagree, N(%) | 352 (75.5) | 68 (76.4) | 40 (38.5) | <0.001 | 0.621 | <0.001 | <0.001 |
| Neutral, N(%) | 52 (11.2) | 12 (13.5) | 19 (19.3) |  |  |  |  |
| Agree, N(%) | 62 (13.3) | 9 (10.1) | 45 (43.3) |  |  |  |  |
| Likert scale, median (IQR) | 1 (1-4) | 1 (1-3) | 6 (3-8) | <0.001 |  |  |  |
|  |  |  |  |  |  |  |  |
| 8. It is acceptable to maintain LST in patients without reasonable chance of surviving outside the ICU | | | | | |  |  |
| Disagree, N(%) | 409 (87.6) | 68 (76.4) | 40 (38.5) | <0.001 | 0.647 | <0.001 | <0.001 |
| Neutral, N(%) | 25 (5.4) | 7 (7.9) | 29 (27.6) |  |  |  |  |
| Agree, N(%) | 33 (7.1) | 6 (6.7) | 32 (30.5) |  |  |  |  |
| Likert scale, median (IQR) | 1 (1-3) | 1 (1-3) | 5 (3-7) | <0.001 |  |  |  |
|  |  |  |  |  |  |  |  |
| 9. Physicians may choose not to offer treatments that they think are not helpful, even without the patient’s knowledge | | | | | | | |
| Disagree, N(%) | 177 (38) | 36 (40.4) | 73 (69.5) | <0.001 | 0.567 | <0.001 | <0.001 |
| Neutral, N(%) | 54 (11.6) | 13 (14.6) | 8 (7.6) |  |  |  |  |
| Agree, N(%) | 235 (50.4) | 40 (44.9) | 24 (22.9) |  |  |  |  |
| Likert scale, median (IQR) | 7 (3-8) | 6 (3-8) | 3 (1-5.5) | <0.001 |  |  |  |
|  |  |  |  |  |  |  |  |
| 10. Discussions about end-of-life increase stress and should not be pursued by healthcare professionals | | | | | | |  |
| Disagree, N(%) | 459 (98.9) | 86 (96.6) | 101 (96.2) | 0.183 | 0.216 | 0.061 | 0.897 |
| Neutral, N(%) | 1 (0.2) | 1 (1.1) | 2 (1.9) |  |  |  |  |
| Agree, N(%) | 4 (0.9) | 2 (2.2) | 2 (1.9) |  |  |  |  |
| Likert scale, median (IQR) | 1 (1-1) | 1 (1-1) | 1 (1-3) | <0.001 |  |  |  |
|  |  |  |  |  |  |  |  |
| 11. Physicians need authorization from the judiciary to make decisions about end-of-life care | | | | | |  |  |
| Disagree, N(%) | 451 (96.8) | 85 (95.5) | 78 (74.3) | <0.001 | 0.782 | <0.001 | <0.001 |
| Neutral, N(%) | 10 (2.1) | 3 (3.4) | 20 (19) |  |  |  |  |
| Agree, N(%) | 5 (1.1) | 1 (1.1) | 7 (6.7) |  |  |  |  |
| Likert scale, median (IQR) | 1 (1-1) | 1 (1-2) | 3 (3-5) | <0.001 |  |  |  |

LST, life sustaining treatment

ICU, intensive care unit
